# The Supporter Needs Supporting Too: A Qualitative Study to Understand the Maternity Care Experience for Partners Following a Previous Perinatal Death

**DOI:** 10.1111/hex.70464

**Published:** 2025-10-16

**Authors:** Debbie M. Smith, Joanna Beaumont, Emilie Bailey, Rebecca Barron, Margaret Murphy, Emma Tomlinson, Alexander E. P. Heazell

**Affiliations:** ^1^ Division of Psychology & Mental Health, School of Health Sciences, Faculty of Biology, Medicine and Health, The Manchester Centre for Health Psychology University of Manchester Manchester UK; ^2^ Maternal and Fetal Health Research Centre, Division of Developmental Biology and Medicine, School of Medical Sciences, Faculty of Biology, Medicine and Health University of Manchester Manchester UK; ^3^ Saint Mary's Hospital Manchester University NHS Foundation Trust Manchester UK; ^4^ School of Nursing and Midwifery University College Cork Cork Ireland

**Keywords:** fathers, partners, pregnancy after loss, stillbirth

## Abstract

**Background:**

Birthing mothers are likely to experience high levels of psychological distress in a pregnancy following a perinatal death. However, less is known about the impact of a subsequent pregnancy after perinatal death on partners and their experiences of maternity care.

**Objective:**

To explore partners’ maternity care experiences and support needs in a pregnancy following a perinatal death.

**Design:**

Qualitative descriptive design.

**Setting and Participants:**

Partners attending a specialist antenatal clinic in a UK tertiary maternity service for families who are pregnant following a previous perinatal death. Twenty‐seven partners were interviewed antenatally and 21 were interviewed postnatally.

**Analysis:**

Data were analysed using inductive thematic analysis.

**Results:**

Two themes described the partners’ experiences: *‘If she's happy, I'm happy’*: Feeling supported’ where partners described taking on the role as the supporter to the birthing mother, and *Guided ‘…out of the shadows’* where partners expressed the need to feel included in maternity care and to have access to support.

**Discussion:**

Maternity care services need to recognise the role of partners as supporters for bereaved mothers, particularly in pregnancy after loss. Many partners do not feel included in maternity care and gender roles encourage men to behave in stereotypical masculine ways which may explain why most do not engage in health seeking behaviour to support their mental health.

**Conclusions:**

Support from specialist healthcare professionals at a dedicated pregnancy after loss clinic helps partners to support the birthing mother. Although partners describe symptoms of psychological distress in a subsequent pregnancy, there are no care pathways that provide psychological support to partners. Greater inclusion in maternity care would impact positively on partners’ experience. Training for healthcare professionals to engage in healthcare behaviours would improve the maternity care experience for partners who have experienced perinatal death.

**Patient or Public Contribution:**

A patient and public involvement (PPI) group for the study included parents who had lived experience of pregnancy after loss. The PPI group advised on study design and the presentation of participant information. Parents who had lived experience of baby loss and who were service users at a specialist antenatal clinic were interviewed for the study.

## 1 Introduction

In 2024, in England and Wales there were 3.9 stillbirths per 1000 total births [1]. The death of a baby can have a devastating impact on both parents, however partners may experience baby loss differently to mothers [2]. Studies have shown that partners adopt the role of the supporter for the birthing mother after pregnancy loss [3, 4, 5, 6, 7, 8] but they lack the support and knowledge to do this when they are also attempting to manage their own suffering [5]. Consequently, partners may hide their emotions after a perinatal death which can have a negative impact on their wellbeing [2, 3, 4, 6, 8]. In addition, they report feeling neglected by healthcare professionals [5, 9]. A recent meta‐synthesis summarising partners experiences after loss showed a lack of support from friends, family and services leading to feelings of isolation, and a reliance on personal coping mechanisms [10].

In a subsequent pregnancy after perinatal death, many parents are likely to experience psychological challenges such as high levels of depression, anxiety, and posttraumatic stress (PTS) [11, 12, 13, 14, 15, 16, 17]. Recent findings show that antenatally approximately one in three partners are likely to be experiencing depression symptoms and one in five partners are likely to be experiencing high levels of anxiety and PTS, [12] and PTS symptoms may continue into the postnatal period [11, 12, 17]. Providing emotional support to families in pregnancies following previous perinatal death was stated in a recent expert review of stillbirth care as ‘more critical’ than medical care [18].

Mothers who attend a specialist pregnancy after perinatal death clinic report feeling emotionally supported as specialist healthcare professionals make them feel safe and understood [19, 20], however, mothers reported that that their partners receive little or no support during a subsequent pregnancy [20]. The need to conduct more research to evaluate the care provided by specialist clinics and clinicians has been proposed in an international prioritisation exercise [21] however, studies must also include partners to identify ways to best support both parents. There are limited qualitative studies that have evaluated partners’ experiences in a subsequent pregnancy following a perinatal death. As such, the current study is the first to explore partners’ experiences of pregnancy following a perinatal death whilst attending a specialist antenatal service for pregnancy after perinatal death. The study aimed to explore partners’ experiences of healthcare and the impact on their wellbeing with a focus on informing future healthcare provisions.

## 2 Methods

### 2.1 Design

This qualitative study was part of a larger mixed‐methods study of partners and birthing mothers [12, 23]. A sample size of 60 partners was calculated to allow for adequate power for the quantitative analysis, all 60 participants in the quantitative study were offered the chance to take part in a qualitative interview. Participants were recruited from the Rainbow Clinic at Saint Mary's Hospital, Manchester. This is a specialist clinic providing maternity care to couples who have experienced a previous stillbirth or perinatal death. The clinic follows the international consensus statement for management and care of pregnancies after perinatal death [23]. Partners were eligible for inclusion if their current pregnancy was under the care of the Rainbow Clinic following a previous stillbirth. Partners were excluded if they were unable to read English, were less than 16 years of age or lacked the capacity to consent. The study was granted ethical approval by The University of Manchester.

### 2.2 Procedure

Potential participants were approached at their 23‐week Rainbow Clinic appointment (which is a fixed point for ultrasound screening in the service). Partners who gave their informed written consent were contacted by email or text message at 28 weeks’ gestation and at 3 months postnatal to ask if they would take part in an interview about their experiences of the current pregnancy and care. The research team designed the interview topic guide with the literature base in mind and with input from a patient participation group. The antenatal interview focused on their experiences in the first and second trimester of pregnancy, and the postnatal interview focused on the third trimester of pregnancy, labour, birth and following birth. Table 1 contains the five sections and example questions. Interviews were conducted on zoom or via phone from November 2022 to June 2024 by two researchers (J.B. and D.M.S.). All participants were asked at the end of the interview how they were feeling after discussing this personal experience to ensure they left the interview in a safe psychological state. They all were also sent a debrief email encouraging them to contact the team or a local support service, if required. The interviews were audio‐recorded until they were anonymously transcribed verbatim. Participants selected a pseudonym to allow for cultural and generation variation, these are used alongside quotes.

**Table 1:** Interview topic guide sections and relevant prompts.

Section topic	Opening question	Example probe questions
1: This pregnancy	*Please can you tell me about this pregnancy?*	Used from sections two and three and general prompts.
2: Your experience of maternal care	*Please can you tell me about the maternity care you and your partner received in this pregnancy?*	*Did you receive support from anywhere outside the hospital?*
		*Was any of this care directly for you as the partner?*
	*(Postnatal: Focus on intrapartum and postnatal care)*.	
		*Postnatal: Please tell us about yours and your partner's care during labour and after the birth?*
3: Your personal experience: Feelings	*Can you tell me about your feelings in this pregnancy?*	*Were these feelings the same for your partner?*
		*Are there any timepoints/events that had an impact on your feelings?*
		*How did you cope, control and/or manage these feelings?*
		*Postnatal: How did you feel during labour/birth/after birth?*
4: Support for feelings	*Please can you tell us what or who has had a positive influence on your emotional wellbeing in this pregnancy?*	*If you felt any negative emotions/feelings, how did you cope with these?*
	*Was there anything that could have further/better supported your emotional wellbeing in this pregnancy?*	*If you felt any positive emotions/feelings, what helped to develop these?*
		*What was your experience of the support? Impact? What? How?*
		*Is there any support that you have not had that you would like?*
5: Anything extra to add?	*Is there anything else that you would like to tell us that you think is important to this study?*	General prompts used.
General prompts:		
*Why? Please tell me more about that. Please expand on that. What do you mean by that? Was this unique to you as the partner?*		

#### 2.2.1 Analysis

We used an inductive approach following Braun and Clarke's six‐stages of reflexive thematic analysis to identify latent level themes [24]. Stages 1–4 of the analysis were conducted independently by the researchers who interviewed participants (D.M.S. and J.B.). Both are mothers, one with experience of early pregnancy death but neither have had clinical or personal experience of stillbirth. Following this, J.B. and D.M.S. met on three occasions to refine the themes (stage five). One further author with clinical experience of stillbirth and the lead obstetrician for the Rainbow Clinic (AEH) was involved with theme contextualisation and discussion in the final two stages of the analysis process.

To further enhance our understanding of support for partners and to make recommendations for future healthcare provisions, a diagram of care needs was produced to show the types of support partners reported using or needing in a pregnancy after perinatal death.

## 3 Results

### 3.1 Participants

Sixty‐eight partners gave their informed written consent and were invited to interview. Twenty‐seven partners were interviewed antenatally, and 21 partners were interviewed postnatally. Nineteen partners completed both antenatal and postnatal interviews. The interviews lasted between 24 and 61 min. The mean antenatal and postnatal interview lengths were 38 min (SD = 10.39) and 44 min (SD = 9.13), respectively. One partner identified as nonbinary, and all other partners reported their gender as male. Partners were aged 30–44 (M = 36.6 years; SD = 4.1). The age of one participant was unknown. The birthing mother of all participants delivered a live baby between 35‐ and 41‐weeks’ gestation. The ethnicity, marital status and pregnancy history of participants is shown in Table 2.

**Table 2:** Ethnicity, marital status and pregnancy history of partners (*n* = 29).

Demographic characteristic	Partners
	*N*
Ethnicity	
White British	26
White–any other White background	1
Black or Black British–African	1
Unknown	1
Marital status	
Married	20
Living with partner	8
Single	1
Parity	
1	3
2	16
≥ 3	9
Unknown	1
Interpregnancy interval (months)	
≤ 12	12
13–36	12
≥ 37	2
Unknown	3
Mode of stillbirth	
Vaginal delivery	24
Emergency caesarean section	3
Unknown	2
Gestation at time of loss (months)	
16–23	12
24–32	7
33–41	8
Unknown	2
Type of loss	
Second trimester loss	10
Antepartum stillbirth	13
Termination for medical reasons	3
Neonatal death	2
Unknown	1
Mode of live birth	
Vaginal delivery	7
Elective caesarean section	10
Emergency caesarean section	4
Unknown	8

### 3.2 Inductive Results

Two themes were developed across the antenatal and postnatal partner interviews; ‘*If she's happy, I'm happy’*: Feeling supported’ and *Guided ‘…out of the shadows’*. They summarise partners’ experiences and the role they felt they had in the maternity journey following a previous perinatal death. Central to their experience was their partner's (the birthing mother's) experience, the influence of the maternity care environment, and other people such as healthcare professionals, friends and family. The two themes are portrayed below with the use of verbatim quotes and pseudonyms.

### 3.3 Theme 1 – ‘If She's Happy, I'm Happy’: Feeling Supported

The wellbeing of the birthing mother was central to all the partners’ experiences and shaped their behaviour throughout pregnancy and following birth. The emotional wellbeing of their partner—the birthing mother—impacted on their own wellbeing; they had greater wellbeing when their partner felt increasingly positive about the pregnancy. As such, they focused on doing and saying things that they hoped would make the birthing mother feel better. One partner said that alongside their wellbeing they needed to consider *‘…how to carry on being a good husband as well’* (Gareth) and another said *‘…know any care that's afforded to her is basically afforded to me…’* (Ben). Two subthemes further describe the support discussed in this theme: *Role as supporter* and *role of specialist healthcare*.

#### 3.3.1 Role as Supporter

All partners talked about taking on the role of the supporter to the birthing mother during the pregnancy following their perinatal death, seeing it as their priority to support her mental health and wellbeing. Most partners felt that support for themselves was second to that of the birthing mother being supported. Many partners expressed the need to *‘shield’* (Daniel) and *‘protect’* (Andrew) the birthing mothers. Some partners described taking an active role in facilitating maternity care to ensure the birthing mother was looked after, particularly when they felt the care was inadequate or their voices were not being heard. When care was seen as good, partners saw the healthcare professionals as people who helped them to fulfil their supporter role.

I mean the main thing again, for most of the pregnancy is just helping [birthing mother's name] with her anxiety.

(John)

It's a fact that the communication isn't there, we know that birth experiences aren't positive. We know that the way you get one is by being engaged and having a partner who will advocate for you.

(Peter)

Several partners mentioned that they could not do anything to improve the outcome or experience of the pregnancy, other than support the birthing mother, as they were not physically pregnant. Due to the lack of physical connection with the pregnancy, partners let the birthing mother lead care, particularly in relation to how and when to give birth as it was the birthing mother who would be physically going through the experience.

I'm just trying to support her…I can't physically do anything else. I'm not. I'm not pregnant. So it's basically making sure she's happy and as less anxious as she can be.

(Ben)

I know you sort of take a back seat but as long as mother and baby are healthy then you're okay.

(Robin)

The role of the partner as the supporter continued after birth and some partners still took the leading role in ensuring the birthing mother received the care she needed. For some partners, returning to work hindered their ability to support the birthing mother at home and they found it stressful to manage their workload alongside family responsibilities. Partners who had a long paternity/parenting leave and supportive workplaces spoke about being able to continue providing emotional support to the mother and sharing the responsibility of parenting which helped them to bond with their baby. The lack of support for parents from maternity services following discharge from hospital required many partners to provide increased support for their partner and also left several feeling unsupported in this role.

I think I was quite lucky as well because I got 6 weeks paternity leave from my company, full pay, which is very privileged I know. So that really helped, it helped a lot.

(Marcus)

I've been able to take kind of time off [work] during the days just give [wife's name] a hand with things. And that support's been great really, really good.

(Daniel)

#### 3.3.2 Role of Specialist Healthcare

As stated above, partners felt the focus of care should be on the birthing mother and baby. They also felt that when the birthing mother was receiving personalised antenatal care from the specialist clinic, it improved her mental health which indirectly supported them. When the couples had a pre‐pregnancy arrangement in place from the specialist clinic to receive specialist care for a subsequent pregnancy, this eased anxiety from the beginning of the pregnancy. In some cases, a bereavement midwife helped to facilitate the maternity care and was a source of support and point of contact for the birthing mother providing care within a complex model which involved parents attending the specialist clinic and/or other hospitals for extra appointments and scans due to the previous loss. Having one person who was a *‘trusted agent’* with *‘ownership’* (Paul) made the process easier to navigate and reduced anxiety.

it's [the care] always aimed at [wife's name] but that's fine, because that's what [wife's name] needs.

(Jon)

from the minute of us saying, we're pregnant…we need you guys [specialist healthcare professionals], kind of thing, we felt very supported straight away. Good communication straightaway so we had numerous ways to sort of contact the team, i.e., phone numbers, a direct email…We were very aware, early on that any additional appointment support, we just ask and we can have them.

(Peter)

The specialist maternity care was described as feeling like the *‘red carpet treatment’* (Marcus). Table 3 contains the aspects of healthcare provision and healthcare professionals’ behaviours which were reported by partners to describe the reasons why they felt supported by specialist antenatal care. When the couples attended appointments at local hospitals outside of the specialist clinic, the NHS was frequently reported to be *‘under pressure’* (Robert); partners often felt like staff still cared but they had limited time and resources to be able to support families emotionally in addition to providing medical support. One partner said, *‘Individuals can be very good but the system's screwed’* (Peter). Electronic notes were generally not deemed favourably as they led to confusion over appointments and the lack of a physical rainbow sticker meant healthcare professionals were sometimes unaware that parents had previously experienced a perinatal death which resulted in them having to explain their situation. There were some instances of continuity of carer where couples were able to see the same midwife or consultant from the previous pregnancy in the current pregnancy, which was appreciated as it meant couples did not have to explain their background. In other cases, partners valued seeing people who understood and were empathetic to their situation.

…you feel like there's somebody who knows your care.

(Simon)

**Table 3:** Reasons why partners felt supported in specialist antenatal care.

Aspects of health care provision	Health care professionals' behaviours
Frequent and flexible appointments to ease anxiety and reassure	Refers to statistics and research when giving advice
Continuity of carer through experience means familiarity	Provide clear explanations in consultations
Availability of staff	Explain well‐detailed scans
Lengthy appointments to allow time to ask questions	Show an awareness of their previous pregnancy so they do not need to repeat their past
Calming and secluded environment of the clinic	Empathetic to their needs and situation
Short wait time before scheduled appointments	Honest in conversations
Detailed birth plans are set including practical and emotional aspects	Specialist expertise is shown

Partners valued the specialist clinic and care throughout their pregnancy, however this specialist care ended when the baby was born. Many partners described their postnatal care as being disjointed (e.g., no continuity of carer) and focused on the mother and baby. A few partners wanted less input from healthcare professionals postnatally, for instance if they were receiving contradictory advice relating to how to care for their baby. A few partners spoke about the need to be treated the same as other parents once the baby was born.

So I think at the beginning that [receiving different breastfeeding advice from healthcare professionals] was stressful and I did kind of want to reduce the amount of input we were getting.

(Charlie)

And they bought [wife's name] a menu, and they looked at [wife's name] like “what would you like to eat?”. And I'm like [laughs], “you know, I'm here as well”. So that's when it's almost became probably just a normal parent, and not parents who had gone through what we had before. So maybe that's a positive thing, or maybe they should still have that caring and that additional support, because it's not over yet. We've not got baby home yet.

(Dave)

### 3.4 Theme 2 –Being Guided ‘Out of the Shadows’

All partners said that maternity care should primarily be focused on the birthing mother and baby. However, this focus often left partners feeling alone in their role as a bereaved partner; they used words such as *‘observer’* (Joe) or *‘bystander’* (Terry) to describe themselves when attending appointments. Two subthemes further describe this experience for partners showing where and how they received support: *Being acknowledged by healthcare professionals* and *seeking own support*.

#### 3.4.1 Being Acknowledged by Healthcare Professionals

To feel they were being guided out of the shadows, partners wanted to be acknowledged by healthcare professionals and included in the maternity care. They felt included at appointments when healthcare professionals involved them in discussions, gave them the opportunity to ask questions, and asked them about their wellbeing. Some partners felt ignored by healthcare professionals as they were not acknowledged at appointments and were not asked about their mental health, particularly when accessing nonspecialist medical care, making them feel unsupported.

I'm never, my opinions are never solicited. I have to volunteer.

(Marcus)

Oh yeah, they care that I'm here and they want me to be here.

(Matt)

Just to be noticed. I'm not just a little person in the corner of the room. It was just very much like “Oh, and how are you”.

(Terry)

In the later stages of pregnancy, many partners expressed that they felt increasingly anxious as the birth was approaching. However, having a birth plan in place and knowing how the birth would proceed eased these anxieties. In addition, having the opportunity to visit the setting (e.g., room, hospital) where the birth would take place and discussing the birth plan with a trusted healthcare professional also gave partners a sense of control. Partners mentioned that when they were given conflicting advice from healthcare professionals about the safest way to deliver their baby, this caused stress and anxiety.

I'm not medical, my wife's not medical, and it's hard to know what to do [whether to deliver baby at 37 or 38 weeks]. You talk to the medical professionals for that expert opinion, and then it differs slightly, and it's only a slight difference. But it's enough to really unsettle you.

(Leigh)

During the birth, partners expressed the importance of being kept informed particularly when the mother and partner were separated. For instance, when putting scrubs on in preparation for the caesarean some partners expressed that they were left alone for a long period of time and this was when they felt most anxious as they did not know what was happening to the mother and baby.

…but you're still sitting there thinking like if there's no news anything could be happening.

(Terry)

Most partners were able to stay overnight after their baby's birth in a private room which was usually put in place by healthcare professionals before the birth (e.g., by the specialist midwife); however, in some cases partners were still asked to leave at night and the partner and/or mother had to subsequently explain their background to ensure that they were able to stay. Most partners expressed that if they had not been allowed to stay overnight after the birth, this would have been incredibly stressful and anxiety‐provoking for both partner and mother.

So it probably didn't help knowing that once she was going to be handed the baby, I was going to have to leave at a certain point. So yeah, you know, again, in an ideal world, I think if someone had gone through what we had been through where we'd had a caesarean and the baby had died, the ability for me to stay there on that first night might have helped.

(Peter)

I think if we hadn't have been given that side room, I think that would have caused a lot of stress, and I know quite often that wouldn't be the case so a lot of times partners would have to go I think in the night because they wouldn't be allowed in the ward.

(Tim)

#### 3.4.2 Seeking Own Support

Many partners mentioned that they needed some professional support during pregnancy and after birth. However, many said that they had not been signposted to support by healthcare professionals, signposting in the hospital was not visible, and they would not know how to access support. During postnatal maternity appointments, many partners did not recall being asked about their wellbeing or signposted to services, however some partners described experiencing anxiety, grief or PTS symptoms in this period.

There's definitely never been a mechanism of, you know, how's dad in that situation. I think one of them actually said to me, “What are you still doing here are you not back at work yet?”

(Matt)

Only a few partners were receiving professional support (e.g., counselling) or were about to commence it privately or through a charity. Partners who felt supported by their friends, family and workplace generally felt that they did not need to take up external avenues of professional support. Some partners mentioned that they did not access support as they were reluctant to talk about their feelings because there was a social *‘taboo’* (Peter) surrounding perinatal death. A few partners said that seeking support was not something that was easy for them as they had been raised to *‘… get on and do’* (Jon), or that perinatal mental health support was aimed at mothers not partners. Some partners were signposted to or approached by a representative from a dad's charity in the specialist clinic waiting room, and partners who were felt reassured that support was there if they needed it: *‘good to have it in their “back pocket”’* (Paul). However, no partners had accessed this support.

…she's (wife) done a few of those sessions, and it's definitely seen a huge improvement into how she's thinking about things. And I tried to look for myself to find something similar, but it all seemed to be aimed at women losing their babies.

(Colin)

Many partners expressed that they would have liked informal mental health support (e.g., chats with a nurse, peer support) rather than formal support (e.g., from a counsellor, psychologist), and some partners expressed they would have liked this support to begin early in the pregnancy. Partners said that they would have liked a one‐to‐one discussion or phone call with a specialist midwife or trusted healthcare professional away from the birthing mother as many expressed that they would not feel comfortable talking openly about their feelings with her present.

…it depends on the relationship, you might not want to open up in front of your partner if it's not the right time.

(John)

For those nine months, I think it's very much about being the rock and the solid bit. You don't want your wife and things sort of seeing you crack a little bit.

(Matt)

Some partners felt that the best way for them to feel supported would be through connecting with another bereaved parent of the same sex as they felt alone in their social worlds. Most partners did not want group support as they would not feel comfortable talking about their feelings within a group setting. A few partners engaged in self‐care (e.g., exercise) to support their mental health and some mentioned drawing on support by using websites or digital media (e.g., Dads Still Standing podcast). Some partners, in the postnatal interviews, said that they would like the opportunity to support other bereaved partners themselves who were going through pregnancy following perinatal death. Many partners felt that support should be offered at different stages in the pregnancy and again after the birth to check‐in on them.

I definitely think if they'd have been a direct, you can go and chat to this person, preferably a man, and say have an open conversation with him and explore my feelings more around issues of being a husband, dealing with a wife, loss, or how to deal with a new pregnancy as a man, or how to manage day to day going back to work after loss, these sorts of things, then I definitely would have gone for that.

(Gareth)

if I was to be asked to go and, you know, buddy up with a dad that's going through the aftermath of a stillbirth now, like I'd love to do that. You know, I'd love to provide that support.

(Stan)

maybe kind of, you know, a bit later on [in the pregnancy], you know, someone checking in later on. And saying, you know, or monthly, or something, just saying, “how are you doing?” kind of thing.

(Robert)

### 3.5 Diagram of Care

**Figure 1:**
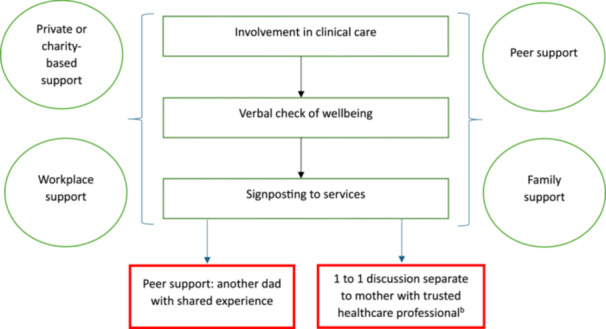
Support needs for partners in a pregnancy following perinatal death.^a^ ^a^The green rectangles represent the steps that need to be taken by healthcare providers to support partners during maternity care appointments. The green circles represent external sources of support that partners may need. The red rectangles represent further support that partners may require if their support needs are not met by those sources in the green rectangles and circles. ^b^Some partners may need one‐to‐one informal conversations with trusted healthcare professionals (e.g., a midwife) at the maternity care appointments, and some may need formal meetings with mental health professionals (e.g., a counsellor, psychologist).

All examples of support mentioned by partners in a subsequent pregnancy following perinatal death were acknowledged and collated to show their experience of healthcare (Figure 1). The diagram shows that to provide support for partners’ healthcare professionals should directly involve them in care, enquire about their wellbeing and signpost to services. In addition, partners are likely to feel supported outside of maternity care if they have support from their family, friends, workplace or are already receiving professional support (e.g., counselling). Partners who do not feel supported by the aforementioned factors will likely need additional support such as further professional support, discussions or check‐ins with a midwife or peer support from another partner with shared experience.

## 4 Discussion

The current findings support previous work showing that partners adopt the role of the supporter for the birthing mother after pregnancy loss [2, 3, 4, 5, 6, 7, 8] and that they continue adhering to their supporter role when embarking on a subsequent pregnancy. The role of partner support for birthing mothers has also been recognised in other maternity‐related behaviours such as breastfeeding [25]. Parallels with our findings and the breastfeeding literature can be seen as many partners in our study felt excluded from care, a lack of recognition of their role, and had little information and advice given directly to them [26]. The need to recognise the role of partners as supporters for bereaved mothers is not a new concept and was concluded as something that needed recognition when planning maternity care to support bereaved families [27].

Male partners face many challenges following a perinatal death due to socially constructed gender roles [3]. Socio‐cultural ideas encourage men to behave in stereotypical masculine ways such as by hiding their feelings [28] and being ‘strong’ for their partner which means concealing their own distress and grief [8]. Men who are suffering from psychological distress may rely on their female partners to provide emotional support in private [29] to maintain their adherence to social norms associated with masculinity (e.g., hiding their emotions) [30]. However, many partners in this study described being unable to rely on the birthing mother for emotional support as she needed to be emotionally supported herself. As such, many partners who are experiencing pregnancy after loss may be hiding their distress and have little or no emotional support during this time which is likely to increase their levels of psychological distress. Indeed, findings from the quantitative (survey) component of this study showed many partners had symptoms of psychological distress (anxiety, depression, and/or PTS) antenatally and postnatally [12].

Bandura's social cognitive theory posits that we learn behaviour through our knowledge of a behaviour, the outcome(s) attributed to a behaviour, and the reaction a behaviour receives from those in the social environment [31]. Partners in this study talked about their own health seeking behaviour as secondary to supporting the birthing mother and thus not something enforced by themselves or healthcare professionals. For most partners, no support was offered and they did not feel included in care by healthcare professionals, however gender roles around them posit that they do not require support so it is not surprising that they did not engage in health seeking behaviour. Considering social cognitive theory, healthcare professionals and maternity care commissioners need to be aware that their behaviours and the wider environment will influence the learned health seeking behaviours of the partner through reinforcement and association.

### 4.1 Strengths and Limitations

Men traditionally have shown less interest in participating in health research than women [32]. Qualitative studies exploring stillbirth have found it hard to recruit males [7] and previous studies with fathers [17] have found low engagement with interviews [33]. It may be difficult to recruit men for qualitative studies as they are more reluctant to speak about their feelings and experiences than women. In this study, partners showed a clear desire to take part in research and inform future care; they were keen to share their stories and many expressed thanks to the research team for listening to them and including them in research. However, our study may have only captured the experiences of partners who felt comfortable expressing their emotions and speaking about their experiences. Partners who hide their feelings may prefer different forms of support compared to partners who are comfortable speaking about their emotions (e.g., text‐message support [34]). As such, a questionnaire‐based study which asks partners about their preferred means of support during a pregnancy following loss may be a means to capture the experiences of partners who are reluctant to engage in qualitative research.

There are few studies which have focused on identifying partners’ needs in pregnancy after loss; studies that do focus on partners’ experiences typically include participants who identify as male. For instance, in this study, there was only one partner who did not identify as being male. Future studies need to ensure that partners who do not identify as men are included to determine if they have the same support needs, and to identify whether findings can be generalised to individuals of any gender identity. As most partners identify as male, support for partners is likely to include signposts to groups for men or dads. For instance, many partners in this study mentioned being signposted to a dad's charity which undoubtedly provides important support to many fathers; however, such support for men is not inclusive for partners who do not identify as being male (e.g., they may identify as female or nonbinary). Support services for partners need to consider how they can appeal to fathers but also be inclusive for all gender identities.

### 4.2 Implications for Healthcare

The current study highlighted a lack of available and accessible care for partners during the antenatal and postnatal periods in a subsequent pregnancy following perinatal death. It also highlights a need to normalise care for partners as partners (who are typically men) are less likely to engage in health‐seeking behaviour (e.g., seek out a counsellor for support) due to socio‐cultural views of gender. It is important to ensure partners are included in the maternity care experience through the behaviours of healthcare professionals, which may include directly acknowledging partners at the beginning of the appointment, giving them the opportunity to speak and ask questions, asking them how they are feeling and actively signposting them to relevant services. In addition, understanding the role of the social context on behaviours can be useful when designing interventions to support behaviours and thus partners should be involved in the design process.

Partners expressed that they would have liked more help to support the birthing mother through their pregnancy. *‘Think family’* is one principle in the best practice guideline for the inclusion of partners in the perinatal mental health support of mothers [35]. This principle encourages healthcare professionals and commissioners to think about the wider family when considering the health of mothers and should be applied into maternity care following perinatal death. Likewise, services and healthcare professionals supporting partners in a pregnancy following a previous loss must acknowledge the impact of the loss and take a trauma‐informed approach (TIA) to care utilising frameworks such as those outlined by Benton et al. [36]. However, this framework was devised following an evidence‐review and the existing evidence had little coproduction or inclusion of partners; more work may be required to understand how to apply a TIA to support partners.

Partners’ needs must be recognised, and healthcare professionals must treat them as individuals in maternity care following a previous loss [2, 37]. The interviews highlighted aspects of healthcare professionals’ behaviours in the delivery of healthcare which impacted on the partners’ experience. For example, healthcare professionals training should enhance their psychological skills, in particular their knowledge about pregnancy after perinatal death and cognitive and interpersonal skills. Enhancing these will change healthcare professionals’ behaviours in healthcare delivery and improve partners’ perceived experiences of support.

## 5 Conclusion

Partners take on the role as supporter for the birthing mother in a subsequent pregnancy following perinatal death, but this may be at detriment to their own wellbeing. Medical and emotional support for the birthing mother delivered by specialist healthcare professionals in a subsequent pregnancy indirectly supports the partner's mental health. Partners would further benefit from support (e.g., informal conversations with trusted healthcare professionals, peer support) specifically for them. Partners describe symptoms of psychological distress (e.g., anxiety, PTS) both antenatally and postnatally however most do not receive professional support or signposting to support services by healthcare professionals. Greater inclusion in maternity care would impact positively on partners’ maternity care experience. Recommendations of how to include partners who have experienced perinatal death in maternity care include training for healthcare professionals to engage in healthcare behaviours.

## Author Contributions

Alexander E.P. Heazell, Debbie M. Smith, Margaret Murphy and Joanna Beaumont contributed to the study design. Alexander E.P. Heazell obtained funding and had overall responsibility for the study. Emilie Bailey, Rebecca Barron, Emma Tomlinson and Alexander E.P. Heazell were responsible for recruiting participants and collecting demographic data. Joanna Beaumont and Debbie M. Smith analysed the data with input from Alexander E.P. Heazell. Joanna Beaumont, Debbie M. Smith, Margaret Murphy and Alexander E.P. Heazell were responsible for the drafting of the manuscript. All authors gave approval for the final version of the manuscript.

## Ethics Statement

The study was granted ethical approval by the Health Research Authority, South Central–Berkshire B Research Ethics Committee (Ref 22/SC/0108).

## Conflicts of Interest

The authors declare no conflicts of interest.

## Data Availability

Due to the sensitive nature of the study participants were not asked to give permission for data sharing. Anonymised, unlinked data, are available from the authors on request.
